# Comparison of toxicity and outcomes of concurrent radiotherapy with carboplatin/paclitaxel or cisplatin/etoposide in stage III non–small cell lung cancer

**DOI:** 10.1002/cam4.142

**Published:** 2013-10-16

**Authors:** Mun Sem Liew, Joseph Sia, Maud H W Starmans, Ali Tafreshi, Sam Harris, Malcolm Feigen, Shane White, Allan Zimet, Philippe Lambin, Paul C Boutros, Paul Mitchell, Thomas John

**Affiliations:** 1 Austin-Ludwig Oncology Unit, Olivia Newton-John Cancer and Wellness Centre, Austin HealthMelbourne, Australia; 2 Ludwig Institute for Cancer Research, Olivia Newton-John Cancer & Wellness Centre, Austin HealthMelbourne, Australia; 3 Department of Medicine, Austin HealthMelbourne, Australia; 4 University of MelbourneMelbourne, Australia; 5 Department of Radiation Oncology, Olivia Newton-John Cancer & Wellness Centre, Austin HealthMelbourne, Australia; 6 Informatics and Biocomputing Platform, Ontario Institute for Cancer ResearchToronto, Canada; 7 Department of Radiation Oncology (Maastro), GROW-School for Oncology and Developmental Biology, Maastricht University Medical CenterMaastricht, the Netherlands

**Keywords:** Carboplatin/paclitaxel, cisplatin/etoposide, concurrent chemoradiotherapy, locally advanced, non–small cell lung cancer, stage III

## Abstract

Concurrent chemoradiotherapy (CCRT) has become the standard of care for patients with unresectable stage III non–small cell lung cancer (NSCLC). The comparative merits of two widely used regimens: carboplatin/paclitaxel (PC) and cisplatin/etoposide (PE), each with concurrent radiotherapy, remain largely undefined. Records for consecutive patients with stage III NSCLC treated with PC or PE and ≥60 Gy chest radiotherapy between 2000 and 2011 were reviewed for outcomes and toxicity. Survival was estimated using the Kaplan–Meier method and Cox modeling with the Wald test. Comparison across groups was done using the student's *t* and chi-squared tests. Seventy-five (PC: 44, PE: 31) patients were analyzed. PC patients were older (median 71 vs. 63 years; *P* = 0.0006). Other characteristics were comparable between groups. With PE, there was significantly increased grade ≥3 neutropenia (39% vs. 14%, *P* = 0.024) and thrombocytopenia (10% vs. 0%, *P* = 0.039). Radiation pneumonitis was more common with PC (66% vs. 38%, *P* = 0.033). Five treatment-related deaths occurred (PC: 3 vs. PE: 2, *P* = 1.000). With a median follow-up of 51.6 months, there were no significant differences in relapse-free survival (median PC 12.0 vs. PE 11.5 months, *P* = 0.700) or overall survival (median PC 20.7 vs. PE 13.7 months; *P* = 0.989). In multivariate analyses, no factors predicted for improved survival for either regimen. PC was more likely to be used in elderly patients. Despite this, PC resulted in significantly less hematological toxicity but achieved similar survival outcomes as PE. PC is an acceptable CCRT regimen, especially in older patients with multiple comorbidities.

## Introduction

Lung cancer accounts for most cancer deaths worldwide, with the incidence in the developing world set to rise [1]. Most patients are diagnosed with a nonresectable disease, and 30–40% are considered locally advanced, comprising both stage IIIA and IIIB.

Concurrent chemoradiotherapy (CCRT) confers a significant improvement in overall survival (OS) when compared to sequential chemotherapy followed by radiotherapy [2, 3], although the incidence of toxicities such as esophagitis, neutropenia, and anemia are higher [4]. The rationale behind the combined modality approach is that radiotherapy provides local tumor eradication while chemotherapy reduces micrometastatic foci and is a radiosensitizer. The reported 5-year survival rates for CCRT, sequential therapy, and radiotherapy alone are 25%, 15%, and less than 10%, respectively [5]. The risk of locoregional relapse and distant metastasis were lower in CCRT compared with radiotherapy alone [4].

There are various concurrent chemotherapy combinations that have been trialed, with most using a platinum compound as a backbone. At present, the optimal CCRT regimen is not clearly defined. Regimens that have been used in phase III studies include mitomycin, vindesine, and cisplatin [2]; etoposide and cisplatin (PE) [3]; vinblastine and cisplatin [3]; paclitaxel and carboplatin (PC) [6]; as well as vinorelbine and cisplatin [7]. No randomized phase III trials, however, have directly compared the different CCRT regimens, although a recent randomized phase II trial directly compared PC versus PE with concurrent chest radiotherapy in a Chinese cohort with unresectable stage III non–small cell lung cancer (NSCLC) [8].

Our institution has adopted the use of either PE or PC as standard for Stage III NSCLC. Treatment preference is largely dictated by age and convenience, with younger patients and those preferring less frequent chemotherapy treated as per the SWOG 9019 protocol. We aimed to retrospectively review the efficacy and toxicity for patients treated at our institution with curative intent radiotherapy combined with either PE administered according to the SWOG 9019 protocol or weekly PC.

## Methods

### Patient population

All patients who consecutively received radical CCRT with either PC or PE at our institution (Austin Health, Melbourne, Australia) between 1 January 2000 and 31 December 2011 were identified from our health information system, pharmacy, and radiotherapy database. Medical records were retrospectively reviewed and staging was determined according to the TNM classification seventh edition [9]. Clinical stage was determined from computed tomography (CT) and/or flurodeoxyglucose (^18^F-FDG) positron emission tomography (PET) scans, and pathological nodal stage from mediastinal or supraclavicular lymph node biopsy. Only patients with histologically confirmed, clinical or pathological stage III NSCLC were included in the study. Patient, tumor, and treatment factors were recorded, and the severity of patient comorbidities at the time of diagnosis of cancer was quantified using the Charlson Comorbidity Index (CCI) [10]. The study was approved by the Austin Health Research Ethics Committee.

### Chemotherapy

The PC group received carboplatin (area under the curve [AUC] 2) and paclitaxel (45 mg/m^2^) administered on days 1, 8, 15, 22, 28, and 35 over a 6-week period [11]. The PE group received 50 mg/m^2^ of cisplatin administered on days 1, 8, 29, and 36, and 50 mg/m^2^/day of etoposide delivered on days 1–5 and 29–33 [12].

### Radiotherapy

All patients underwent CT planning for three-dimensional conformal radiotherapy with 6 MV linear accelerator photon beams. Where available, diagnostic PET images were fused with the planning CT to help target delineation. Radiotherapy dose prescriptions ranged from 50 Gy to 70 Gy in 2 Gy per fraction, five fractions a week. Most patients (92%) were prescribed 60 Gy. Two patients were prescribed less than 60 Gy because tumor volumes were deemed too large to meet dose constraints. The gross tumor volume (GTV) included the primary disease and any involved regional lymph nodes. Expansions to create clinical target volumes (CTV) and planning target volumes (PTV) were based on the treating radiation oncologist's preference. The lung dose constraint was specified such that no more than 35% of the pulmonary parenchyma (defined as total lung volume minus PTV) received ≥20 Gy. The maximum point dose allowed for the spinal cord was 50 Gy.

### Toxicity

Toxicities were graded according to the National Cancer Institute Common Toxicity Criteria Adverse Events (CTCAE) version 4.0.

### Follow-up and survival data

The follow-up protocol varied between treating physicians. Typically patients were followed up every three to 6 months during the first 2 to 3 years; and 6 months or annually thereafter. The frequency of repeat surveillance CTs and PET were at the physicians' discretion. The last follow-up was defined as the most recent visit to the clinic. Relapse-free survival (RFS) was defined as the time from the start of CCRT to the first date of disease recurrence on imaging or biopsy; patients without relapse were censored at the last date of follow-up. Local recurrence was defined as any tumor regrowth in hilar, mediastinal, or supraclavicular nodes ipsilateral to the primary site of tumor. OS was defined as the time from the start of CCRT to death from any cause or last follow-up date. Dates and causes of death were retrieved from the medical records and death certificates.

### Statistical analysis

All statistical analyses were performed in R statistical environment (v2.15.2). Differences in patient demographics between PC- and PE-treated patients were assessed with chi-squared tests and two-sided Student's *t*-tests. Further differences in RFS and OS were assessed with Kaplan–Meier survival curves and both univariate and multivariate Cox proportional hazard ratio (HR) modeling analyses followed by the Wald test (survival package v2.37-4). A *P*-value <0.05 was considered significant.

## Results

### Clinicopathological findings

Eighty-three patients were identified from our database. Of these, eight patients were excluded from analysis: two patients had excised solitary brain metastases and six patients did not receive the conventional chemotherapy doses. Seventy-five patients were subsequently included for further analyses. The patients in the PC group were significantly older with a median age of 71 years (range, 44–83) versus 63 years (32–76; *P* = 0.0006). There was no difference in clinical stage and histology for patients who received PC and PE. Other known prognostic variables such as weight loss, Eastern Cooperative Oncology Group (ECOG) performance status, comorbidities, and forced expiratory volume at 1 sec (FEV1) were comparable in both groups (Table 1).

**Table 1:** Clinical and pathology characteristics of the 75 study patients

Characteristics	PC (*n*=44)	PE (*n*=31)	
	*n* (%)	*n* (%)	*P*_*χ*2_/*P*_*t*_
Age (median [range])	71 [44–83]	63 [32–76]	0.0006
Sex			
Male	35 (80)	20 (65)	0.236
Smoking status			
Current	13 (30)	15 (48)	0.234
Former	28 (64)	15 (48)	
Never	3 (7)	1 (3)	
ECOG			0.232
0	8 (18)	11 (35)	
1	33 (75)	18 (58)	
2	3 (7)	2 (6)	
Charlson morbidity index (median [range])	3 [2–6]	2 [2–5]	0.099
Weight loss (>5%)	7 (16)	10 (32)	0.166
FEV1 (median [range])	1.91 [0.78–3.1]	1.85 [0.79–3.1]	0.896
TLCO (median [range])	15.8 [6.79–28.3]	15.1 [3.32–30.1]	0.775
Histology			0.548
Squamous	20 (45)	11 (35)	
Adenocarcinoma	19 (43)	12 (39)	
Large cell	3 (7)	5 (16)	
Other	2 (5)	2 (6)	
Stage			0.128
3A	34 (77)	18 (58)	
3B	10 (23)	13 (42)	

*n*, number; *P*_*χ*2_, chi-squared test; *P*_*t*_, student *t*-test; ECOG, Eastern Cooperative Oncology Group; FEV1, force expiratory volume at 1sec; TLCO, transfer factor of the lung for carbon monoxide; PC, carboplatin/paclitaxel; PE, cisplatin/etoposide.

### Staging

All patients underwent a staging CT scan. PET imaging was performed in 73 of 75 (97%) patients at diagnosis. Fifty-four patients had clinical stage N2 and N3 on imaging. Of these, biopsy confirmation was undertaken in 27 (50%) patients. Confirmation of mediastinal node involvement was undertaken via mediastinoscopy in six cases, endobronchial ultrasound (EBUS) transbronchial biopsy in eleven cases, and thoracotomy in five cases. Supraclavicular node biopsy was performed in five cases.

### Treatment delivery

The median dose of radiotherapy received in both groups was 60 Gy with a mean of 58.3 Gy in the PC group and 58.6 Gy in the PE group. Fifty percent (22 of 44) and 58% (18 of 31) of patients completed the prescribed course of CCRT in the PC and PE groups, respectively. The relative mean dose intensities of chemotherapy were comparable in both the PC and PE groups (carboplatin [90%] and paclitaxel [89%] vs. cisplatin [84%] and etoposide [86%]). The most common reasons for not completing the planned CCRT were radiation esophagitis (nine in PC vs. four in PE), chest infection (four in PC vs. six in PE), febrile neutropenia (four in PC vs. one in PE), and hematological toxicities (four in PC vs. three in PE).

Consolidation chemotherapy was given to five patients in the PE group but none in the PC group (*P* = 0.022). Of these five patients, one received docetaxel, another carboplatin/gemcitabine, and three Stimuvax^**®**^ (Darmstadt, Germany) or placebo as part of the phase III START study (NCT00409188).

### Toxicity evaluation

Five (three in PC vs. two in PE, *P* = 1.000) patients died from the treatment. Of these, two patients died as a consequence of chest infection, one died from pneumonitis, and two died from acute coronary syndromes. Treatment-related toxicities are presented in Table 2. The incidence of all grades pneumonitis was more common in the PC group (*P* = 0.033). The PE group had higher rates of neutropenia and thrombocytopenia (*P* = 0.024 and 0.039, respectively).

**Table 2:** Nonhematological and hematological adverse events, by grade (CTCAE 4.0)

Adverse events	PC (*n*=44)	PE (*n*=31)	
	*n* (%)	*n* (%)	*P*_*χ*2_
Esophagitis			0.151
0	7 (16)	8 (26)	
1	3 (7)	5 (16)	
2	19 (43)	7 (23)	
3	10 (23)	10 (32)	
4	5 (11)	1 (3)	
Pneumonitis			0.033
0	15 (34)	19 (62)	
1	21 (48)	4 (13)	
2	6 (14)	6 (19)	
3	0 (0)	1 (3)	
4	1 (2)	1 (3)	
5	1 (2)	0 (0)	
Neuropathy			0.485
0	42 (96)	0 (0)	
1	1 (2)	0 (0)	
2	1 (2)	0 (0)	
Nephropathy			0.314
0	41 (93)	26 (84)	
1	3 (7)	4 (13)	
2	0 (0)	0 (0)	
3	0 (0)	1 (3)	
Nausea/Vomiting			0.291
0	29 (66)	21 (68)	
1	7 (16)	7 (23)	
2	8 (18)	2 (6)	
3	0 (0)	1 (3)	
Chest infection			0.534
0	29 (67)	20 (65)	
1	1 (2)	0 (0)	
2	1 (2)	3 (10)	
3	11 (25)	5 (16)	
4	1 (2)	2 (6)	
5	1 (2)	1 (3)	
Neutropenia			0.024
0	29 (66)	17 (55)	
1	4 (9)	2 (6)	
2	5 (11)	0 (0)	
3	6 (14)	8 (26)	
4	0 (0)	4 (13)	
Febrile neutropenia			0.394
0	39 (89)	25 (81)	
3	5 (11)	5 (16)	
4	0 (0)	1 (3)	
Anemia			0.117
0	26 (60)	11 (36)	
1	12 (27)	10 (32)	
2	5 (11)	9 (29)	
3	1 (2)	0 (0)	
4	0 (0)	1 (3)	
Thrombocytopenia			0.039
0	40 (91)	23 (74)	
1	1 (2)	4 (13)	
2	3 (7)	1 (3)	
3	0 (0)	3 (10)	
Treatment-related deaths	3 (7)	2 (6)	1.000

*n*, number; *P*_*χ*2_, chi-squared test; PC, carboplatin/paclitaxel; PE, cisplatin/etoposide.

### Survival and response

**Figure 1:**
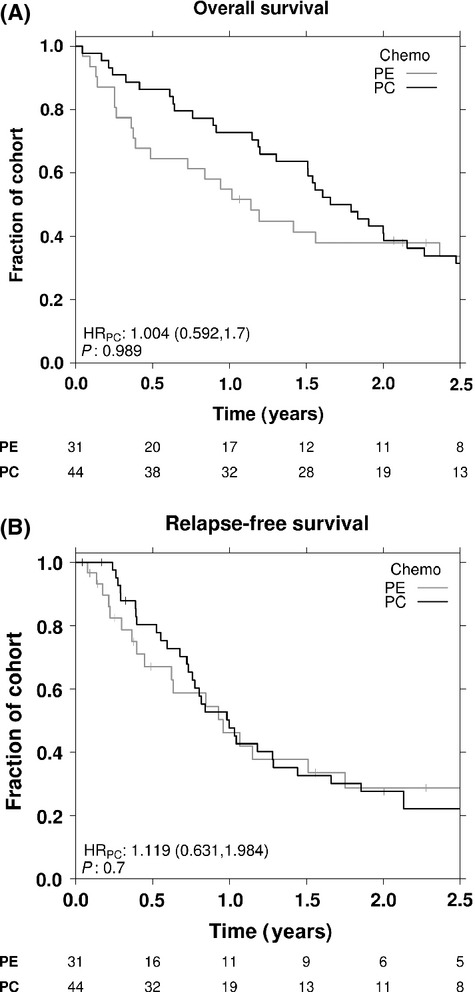
(A) Overall survival and (B) relapse-free survival Kaplan–Meier curves. *x*-axis is truncated at 2.5 years given limited numbers at risk beyond this point.

With a median follow-up of 51.6 months, the median OS for all patients was 18.7 months (95% CI: 14.2–25.9). Sixty (80%) patients had died at the time of data collection. Patients receiving PC did do better than patients receiving PE within 2 years for OS. The median OS favored the PC group, although this was not significant (PC 20.7 months vs. PE 13.7 months, *P* = 0.989) (Fig. 1). Age and consolidation chemotherapy were the only two variables statistically different between the PC and PE group. When adjusted for these two variables, there was no difference in OS between groups (HR 0.99, 95% CI 0.54–1.83, *P* = 0.983).

Fifty-two (69%) patients had relapsed disease. The median RFS was 12 months in the PC group and 11.5 months in the PE group, which was not significant (HR 1.12, 95% CI 0.63–1.98, *P* = 0.700) (Fig. 1). Locoregional, contralateral relapses, and distant metastases were observed in 34 (45%), 16 (21%), and 47 (63%) patients, respectively. Among the 47 patients who had distant relapse, bone metastases were observed in 16 (34%) patients and were the most frequent site of distant metastases. This was followed by liver (*n* = 13, 28%), brain (*n* = 12, 26%), and adrenal (*n* = 4, 9%). There were no differences in pattern of relapse between both groups. Twenty-three (44%) (15 in PC and 8 in PE group) patients received palliative chemotherapy following progression.

In multivariate analysis, age, sex, smoking status, performance status, comorbidities, histology, stage, type of chemotherapy regimen, consolidation treatment, and completion of CCRT were not significantly prognostic for RFS or OS (Table 3).

**Table 3:** Multivariate analyses of association between covariates and relapse-free survival and overall survival

	Overall survival			Relapse-free survival		
		
Variables	HR	95% CI	*P* (Wald test)	HR	95% CI	*P* (Wald test)
Age	1.03	0.99–1.06	0.117	1.00	0.97–1.03	0.974
Sex (vs. female)	1.00	0.47–2.14	1.000	0.44	0.19–1.05	0.066
Smoking status (vs. current)						
Former/Never	0.75	0.41–1.38	0.355	0.84	0.43–1.67	0.624
ECOG (vs. 0)						
1/2	0.92	0.45–1.90	0.826	0.63	0.31–1.30	0.210
Charlson morbidity index	0.90	0.64–1.27	0.566	1.06	0.76–1.50	0.722
Histology (vs. squamous)						
Nonsquamous	0.82	0.44–1.52	0.527	1.27	0.66–2.46	0.474
Stage (vs. 3A)	1.15	0.58–2.28	0.681	1.11	0.52–2.36	0.785
Chemo schedule (vs. PE)	0.87	0.47–1.59	0.643	1.38	0.71–2.69	0.338
Completed scheduled CCRT	0.66	0.35–1.26	0.208	1.32	0.66–2.63	0.439

HR, hazard ratio; CI, confidence interval; CCRT, concurrent chemoradiotherapy; PC, carboplatin/paclitaxel; PE, cisplatin/etoposide.

## Discussion

In this single institution retrospective study, we compared the toxicity and outcomes of PC and PE with concurrent chest radiotherapy in patients with unresectable stage III NSCLC. We found that PC was more likely to be used in elderly patients but resulted in significantly less hematological toxicity but a higher risk of radiation pneumonitis. Despite this, the PC group achieved similar survival outcomes compared to PE.

Most cancer centers use taxanes or etoposide in combination with a platinum agent and concurrent chest radiotherapy to definitively treat unresectable stage III NSCLC. The median OS in prospective clinical trials using these regimes ranges from 16 to 22 months for PC [11, 13, 14], and 15 to 26 months for PE [3, 12, 15–17] (Table 4). The patients' median age in these studies was around 63 years, which is considerably younger than our study of 67 years for all patients and 71 years in the PC group. Our patient cohort comprised “real-world” non-trial patients, and it was therefore reassuring to demonstrate similar survival outcomes to that previously reported in clinical trials.

**Table 4:** Summary of phase II/III trial results for CCRT with PE and PC regimes for inoperable stage III NSCLC

Trial	Pts (*n*)	Median age (years)	Treatment schedule	Response	Median OS (months) (95% CI)	Survival%	Toxicity
Curran etal. [3] phase III	187	62	Arm 3: PE+hypofractionated RT 69.6Gy	CR 33%	15.6 (13.0–18.0)	5years OS: 13%	Gr≥3 neutropenia 54% Gr≥3 esophagitis 45% Gr≥3 pulmonary toxicity: 17%
Belani etal. [11] phase II	74	NR	Arm 2: induction PC followed by concurrent PC+RT 63Gy	NR	12.7 (NR)	3years OS: 15%	Arm 2: Gr≥3 neutropenia 16% Gr≥3 esophagitis 19% Gr≥3 pneumonitis 4%
	92		Arm 3: PC+RT 63Gy followed by consolidation PC		16.3 (NR) (*P-*values NR)	3years OS: 17%	Arm 3: Gr≥3 neutropenia 26% Gr≥3 esophagitis 28% Gr≥3 pneumonitis 16%
Albain etal. [12] phase II	50	58	PE+RT 61Gy	NR	15 (11–22)	5years OS 15%	Gr 4 neutropenia 32% Gr≥3 esophagitis 20% Gr≥2 pneumonitis 0%
Yamamoto etal. [13] phase III	147	63	Arm 3: PC+RT followed by consolidation PC	Arm 3: CR 3.4%, PR 59.9%, SD 21.8%, PD 10.9%	22.0 (NR)	5years OS 19.5%	Arm 3: Gr≥3 neutropenia 61.9% Gr≥3 esophagitis 8.2% Gr≥3 pneumonitis 4.1% (3 deaths)
Vokes etal. [14] phase III	182	63	Arm 1: PC+RT 66Gy	NR	12 (NR)	3years OS 19%	Arm 1: Gr≥3 neutropenia 15% Gr≥3 esophagitis 32% Gr≥3 pneumonitis 4%
	184		Arm 2: induction PC followed by PC+RT 66Gy		14 (NR) (*P*=0.3)	3years OS 23%	Arm 2: Gr≥3 neutropenia 31% Gr≥3 esophagitis 30% Gr≥3 pneumonitis 10%
Gandara etal. [15] phase II	83	60	PE+RT 61Gy followed by consolidation docetaxel	CR 7% PR 60% SD 23% PD 10%	26 (18–35)	3years OS 37%	Gr≥3 neutropenia 74% Gr≥3 esophagitis 17% Gr≥3 pneumonitis 7% (2 deaths)
Hanna etal. [16] phase III	73	62	Arm 1: PE+RT 59.4Gy	NR	23.2 (NR)	3years OS 26.1%	Arm 1: Gr≥3 neutropenia 32% Gr≥3 esophagitis 17.2% Gr≥3 pneumonitis 1.4%
	74		Arm 2: PE+RT 59.4Gy followed by consolidation docetaxel		21.2 (NR) (*P*=0.883)	3years OS 27.1%	Arm 2: Gr≥3 neutropenia 24.7% Gr≥3 esophagitis 17.2% Gr≥3 pneumonitis 9.6%
Kelly etal. [17] phase III	118	61	Arm1: PE+RT 61Gy followed by consolidation docetaxel	NR	35 (NR)	2years OS 59%	For both arms (prior to randomization): Gr≥3 neutropenia 43%
	125		Arm2: PE+RT 61Gy followed by consolidation docetaxel followed by maintenance gefitinib		23 (NR)	2years OS 46%	Gr≥3 esophagitis 14% Gr≥3 pneumonitis 7% (1 death)

Pts, patients; PC, carboplatin/paclitaxel; PE, cisplatinum/etoposide; MVP, mitomycin/vindesine/cisplatinum; RT, radiotherapy; CR, complete response; PR, partial response; SD, stable disease; PD, progressive disease; OS, overall survival; NR, not reported.

In the United States, the median age at diagnosis of lung cancer is 70 years [18] compared with 72 in Australia [19]. Elderly cancer patients may have a relatively lower tolerance of chemotherapy because of underlying comorbidities. This often imposes more medical and physiological challenges that make the selection of the cytotoxic agent more difficult. Evidence from randomized controlled trials focused specifically on older patients is lacking for patients treated with CCRT. The pivotal Japan Clinical Oncology Group (JCOG) 0301 trial [20] provided evidence that single agent carboplatin with concurrent 60 Gy radiotherapy is well tolerated and leads to better survival than radiotherapy alone (HR 0.68, 95% CI 0.47–0.98, *P* = 0.0179) in patients older than 70 years with unresectable stage III NSCLC. As in most prospective clinical trials, the JCOG 0301 was restricted to elderly patients with good performance status, limited comorbidities, and stable organ function. Some retrospective data have also concluded that CCRT is feasible and improves OS and RFS in elderly but fit patients with acceptable toxicity [21–23].

Comorbidities in elderly patients with lung cancer are a prognostic factor for survival and a risk factor for complications with chemotherapy [22, 24, 25]. Korean investigators reviewed 125 patients aged ≥70 years receiving radical radiotherapy with or without concurrent chemotherapy for stage III NSCLC and demonstrated that cardiovascular dysfunction (HR 2.10, 95% CI 1.01–4.39, *P* = 0.048) and a simplified comorbidity score (SCS) of ≥10 (HR 1.55, 95% CI 1.16–2.09, *P* = 0.003) were independent prognostic factors for poor survival [25]. There is no consensus regarding the optimal comorbidity stratification tool for geriatric assessment. The SCS [26] and CCI [10] are simple and time-effective assessment tools for cancer patients. Other scoring systems exist, but the ultimate aim is to ascertain if the patient is to be regarded as “fit elderly” or “frail elderly” to guide more aggressive radical treatment. Whether these indices alter outcome in patients treated with CCRT or whether they can be used to determine patient suitability has yet to be demonstrated in prospective studies.

The PC regimen in our study had a higher incidence of fatal pneumonitis (two patients in PC vs. none in PE), although the rates for symptomatic pneumonitis (CTCAE grades 2–4) were lower than the PE group (18% in PC vs. 25% in PE). Symptomatic radiation pneumonitis is a clinically important toxicity, occurring in 15–40% of patients receiving CCRT for NSCLC [27]. Deaths due to pneumonitis must be viewed seriously but the potential confounders inevitable in a retrospective study make definite associations with the type of chemotherapy regimen difficult. A retrospective study reported a challenge to confirm the diagnosis of radiation pneumonitis in 48% of cases due to presence of the confounding factors such as exacerbation of chronic airway disease, chest infection, and tumor progression [28]. Nonetheless, a recent meta-analysis reported that elderly patients who undergo CCRT with PC are at higher risk of pneumonitis compared to PE with an odds ratio 3.33 (*P* < 0.001) [29]. Wang et al. [8] also showed a higher rate of grade ≥2 pneumonitis in the PC group compared to PE (48.5% vs. 25%, *P* = 0.09). Given this increased rate of pneumonitis it appears pertinent to select patients carefully when offering PC.

The increased hematological toxicity in patients treated with PE also warrants review. In the metastatic NSCLC setting, Belani et al. [30] reported PE having higher myelosuppression rates than the PC regimen. Our finding also has demonstrated grade ≥3 neutropenia (39% vs. 14%) and thrombocytopenia (10% vs. none) to be significantly more prevalent in PE regimen. Considering the toxicity profile and the similar survival outcomes, the PC regimen with concurrent chest radiotherapy is a potentially feasible treatment option in carefully selected elderly patients despite the presence of multiple comorbidities.

The recent study by Wang et al. [8]. has many similarities with ours, yet showed different results. Their study demonstrated a significantly improved survival in Chinese patients treated with concurrent radiotherapy with PE regimen over PC (20.2 months in PE vs. 13.5 months in PC, *P* = 0.04). There was an imbalance in patient characteristics between the PC and PE groups in their study such that the PC group had more adverse prognostic characteristics including weight loss, age, and anemia, although these differences did not reach statistical significance. More patients in the PE group received the standard dose of radiotherapy (≥60 Gy) and consolidation chemotherapy, which could also potentially imbalance the favorable survival outcome seen in this study [8].

Despite the survival difference observed by Wang et al. [8], the PC regimen still remains widely used both in clinical practice and also as a comparator group in clinical trials. Firstly, the PC regimen has repeatedly shown similar efficacy and better tolerability when compared to the PE regimen. The recent Radiation Thoracic Oncology Group (RTOG) 0617 study[6] demonstrated a median survival for Stage III NSCLC patients receiving thoracic radiotherapy and concurrent weekly PC with or without cetuximab of 20.7 months, which was better than the vinblastine/cisplatin (17 months) and cisplatin/oral etoposide (15.6 months) regimens used in the preceding RTOG 9410 study [3]. In the setting of palliative chemotherapy for metastatic NSCLC, Belani et al. [30] reported similar efficacy and outcomes between PC and PE, but patients receiving the third generation PC regimen had lower toxicities and better quality of life. Similarly in the neoadjuvant context, Machtay et al. [31] showed that PC, when compared to PE, achieved similar pathological response rates and survival outcomes in patients with locally advanced stage III NSCLC treated with CCRT, but the PC regimen was associated with less grade 3 gastrointestinal toxicities (3% vs. 27%, *P* = 0.02). Secondly, tumor biology in Asian and non-Asian populations is different. In the last decade, there has been considerable interest on the identification of patients with activating epidermal growth factor receptor mutations (*EGFR*_*mut*_). The presence of an *EGFR*_*mut*_ is not only favorably prognostic but also predictive for progression-free survival and increased control rates when treated with tyrosine kinase inhibitors but also chemotherapy [32]. In the setting of radiotherapy, patients with *EGFR*_*mut*_ were also found to have more radiosensitive tumors and had decreased local recurrence rates [33]. The frequency of *EGFR*_*mut*_ in Caucasian populations is approximately 15% while the rate is reported to be much higher, up to 50%, in Asian patients [34].

Our study has several limitations. The retrospective nature does not allow for accurate quantification of the severity of the toxicities and does not capture subjective toxicities including lethargy and neuropathy. Nevertheless, the grading for pneumonitis, hematological, and esophagitis toxicities and other serious adverse events were robust because of regular clinician and dietitian follow-up and routine blood tests before each session of chemotherapy. Secondly, the number of patients was relatively small, thus limiting the analysis of prognostic and predictive factors. Thirdly, the posttreatment follow-up protocol varied between patients. Consequently, the relapse-free interval may be overappreciated, although OS was accurate given the lost to follow-up rate in this study was relatively low (four patients) in this study.

In conclusion, we report on the toxicities and clinical outcomes of patients with unresectable stage III NSCLC treated with concurrent chest radiotherapy with either PC or PE. Given that PC was used predominantly in older patients but resulted in less toxicities but equivalent survival, we believe PC warrants further investigation in randomized studies involving older patients. However, the poor OS with both treatments requires better strategies to improve clinical outcome of patients with unresectable locally advanced NSCLC.
